# Morphology and Chemical Messenger Regulation of Echinoderm Muscles

**DOI:** 10.3390/biology12101349

**Published:** 2023-10-20

**Authors:** Huachen Liu, Muyan Chen

**Affiliations:** The Key Laboratory of Mariculture, Ministry of Education, Ocean University of China, Qingdao 266003, China; liuhuachen@stu.ouc.edu.cn

**Keywords:** echinoderm muscle, myoepithelium, muscle bundle, neurotransmitter, neuropeptide

## Abstract

**Simple Summary:**

Various physiological activities of organisms, including movement, feeding, reproduction, breathing, excretion, etc., all require the participation of their neuromuscular systems. Echinoderms, a phylum closely related to chordates, possess a well-differentiated but simpler muscular system, which provides a great opportunity to trace the evolutionary origins of the vertebrate muscular system. Here, we review the morphology of different musculatures and the effects of different neurotransmitters and neuropeptides involved in muscle regulation in echinoderms. In addition, we highlight the potential molecular mechanisms underpinning the action of these chemical messengers on echinoderm muscles.

**Abstract:**

The muscular systems of echinoderms play important roles in various physiological and behavioral processes, including feeding, reproduction, movement, respiration, and excretion. Like vertebrates, echinoderm muscle systems can be subdivided into two major divisions, somatic and visceral musculature. The former usually has a myoepithelial organization, while the latter contains muscle bundles formed by the aggregation of myocytes. Neurons and their processes are also detected between these myoepithelial cells and myocytes, which are capable of releasing a variety of neurotransmitters and neuropeptides to regulate muscle activity. Although many studies have reported the pharmacological effects of these chemical messengers on various muscles of echinoderms, there has been limited research on their receptors and their signaling pathways. The muscle physiology of echinoderms is similar to that of chordates, both of which have the deuterostome mode of development. Studies of muscle regulation in echinoderms can provide new insights into the evolution of myoregulatory systems in deuterostomes.

## 1. Introduction

Echinoderm, originating from the Greek for “spiny skin”, is a group of marine benthic organisms. As a phylum of deuterostomate invertebrates, echinoderms form a sister clade to the chordates with hemichordates and xenoturbellids [1,2,3]. Their unique evolutionary status and close relationship to chordates, particularly when compared with other invertebrates, make them attractive as upcoming model systems. Extant echinoderms comprise five well-defined classes: Crinoidea, Asteroidea, Ophiuroidea, Holothuroidea, and Echinoidea [4]. Their biological behaviors have a great influence on the physico-chemical processes of submarine ecosystems, including maintaining and improving sediment health, the recycling of nutrients, biomass regulation, and so on [5,6]. With reference to morphology, adult echinoderms show several distinctive characteristics, such as a (usually) pentaradially symmetrical body structure, unique water vascular system, calcium carbonate endoskeleton, and mutable connective tissue [7], and, with reference to physiology, regeneration, autotomy, aestivation, and evisceration [8,9,10,11]. 

Echinoderms possess well-differentiated but simpler muscular systems in comparison with vertebrates, which provide a great opportunity to trace the evolutionary origins of the vertebrate muscular system [12,13]. Like vertebrates, echinoderm muscle systems can be subdivided into two major divisions, somatic and visceral musculature. However, in echinoderms, there seem to be few distinctive differences between these two muscle categories [14]. In terms of its microscopic structure, echinoderm muscle resembles more vertebrate smooth muscle than striated muscle, including body wall muscles, digestive tract muscles, appendage muscles, and arm muscles [14,15,16]. In terms of function, the muscular systems of echinoderms play important roles in various physiological and behavioral processes, including feeding, reproduction, movement, respiration, and excretion [17,18,19,20].

The muscular system of organisms and its innervation together form the neuromuscular system. There are many neurotransmitters in this system involved in regulating muscle activities such as cholines (e.g., acetylcholine, ACh), bioamines (e.g., 5-hydroxytryptamine, 5-HT), amino acids (e.g., L-glutamate), gases (e.g., nitric oxide, NO), and neuropeptides [15,21,22,23]. In vertebrates, the excitation–contraction coupling mechanism of muscles has been studied in detail, including the morphological basis of muscle fibers, action potential conduction, and intracellular Ca^2+^ elevation mechanism [24]. However, little research has been conducted on neuromuscular systems in marine invertebrates including echinoderms. Elphick and Melarange (2001) reviewed the neural control of muscle relaxation in echinoderms and found that it is regulated by at least two parallel neuronal signaling systems [15]. Subsequently, Hill (2001) reviewed the role of Ca^2+^ in muscle contraction in echinoderms, including Ca^2+^-dependent muscle contraction and Ca^2+^ storage and release sites [25]. Recently, pharmacological experiments have led to the discovery of an increasing number of myoactive neuropeptides [23,26]. However, the mechanism of muscle contraction of echinoderms has only been briefly studied at the tissue level, while the signaling pathway at the cellular and molecular levels is not well understood [27]. Here, we comprehensively review the morphology and function of different muscles in echinoderms, as well as the action characteristics and mechanisms of neurotransmitters and neuropeptides that act on these muscles.

## 2. Morphology and Function of Musculature in Echinoderms

Echinoderm muscles occur in many different anatomical locations, including the body wall, digestive system, reproductive system, respiratory system, and water vascular system, but they have a similar histological structure [14]. As in vertebrates, the musculature of echinoderms can be divided into visceral and somatic musculature. Visceral musculature is generally considered to have a myoepithelial organization and somatic musculature is considered to be the large muscles of the body, which consist mainly of muscle bundles containing many myocytes [14,15].

### 2.1. Visceral Musculature

**Figure 1:**
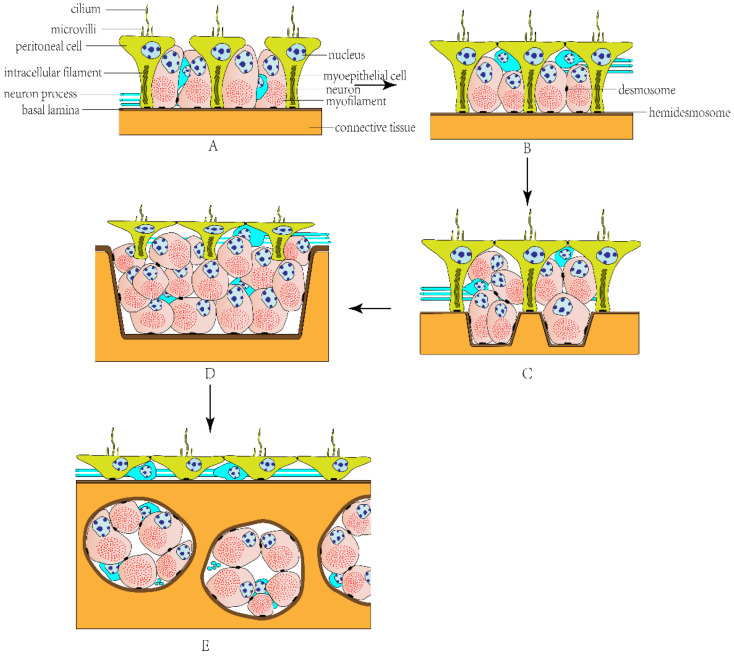
The evolutionary model of echinoderm myoepithelial organization. (**A**) Simple myoepithelium. (**B**) Pseudostratified myoepithelium. (**C**) Bipartite pseudostratified myoepithelium. (**D**) Stratified myoepithelium. (**E**) Subepithelial musculature.

The visceral musculature of echinoderms is the myoepithelium covering celomic surfaces of the internal organs and lining the inner surfaces of the water vascular system, including the musculature of the digestive [28], reproductive [29], and respiratory [30] systems as well as the musculature of tube feet and tentacles [31]. The myoepithelium is mainly composed of myoepithelial and peritoneal cells [14]. Typically, peritoneal cells consist of an extended apical part facing the celomic cavity and a slender basal peduncle penetrating the entire myoepithelium and adhering to the basal lamina through hemidesmosomes. The apical part has an irregularly shaped nucleus, a cilium, and microvilli, and the slender basal peduncle has bundles of intracellular filaments. Myoepithelial cells have a large number of densely packed myofilaments in the cytoplasm, which form a powerful contractile apparatus. They are connected to the basal lamina through hemidesmosomes and to other myoepithelial cells through desmosomes (Figure 1A–E). Myoepithelial cells can be arranged in different directions in different organs, forming oblique, longitudinal, or circular musculature. There are also neurons and their processes between peritoneal and myoepithelial cells, whereas there is no synaptic specialization at the neuron–muscle junction [14,31].

In general, the myoepithelium of the echinoderm visceral musculature is divided into the following types: (1) Simple myoepithelium (Figure 1A): a single-layered myoepithelium; most myoepithelial cells are incorporated into the adluminal surface between peritoneal cells. (2) Pseudostratified myoepithelium (Figure 1B): peritoneal cells are situated apically and myoepithelial cells are situated subapically; not all cells contribute to the adluminal surface, but all cells adhere to the common basal matrix. (3) Bipartite pseudostratified myoepithelium (Figure 1C): myoepithelial cells are either directly anchored to the basal matrix or suspended between the apical and basal surfaces of the myoepithelium. (4) Stratified myoepithelium (Figure 1D): all peritoneal cells lose contact with the basal lamina completely. (5) Subepithelial musculature (Figure 1E): myocytes are below the basal matrix and separated from the celomic lining, forming muscle bundles [31].

#### 2.1.1. Musculature of the Water Vascular System

**Figure 2:**
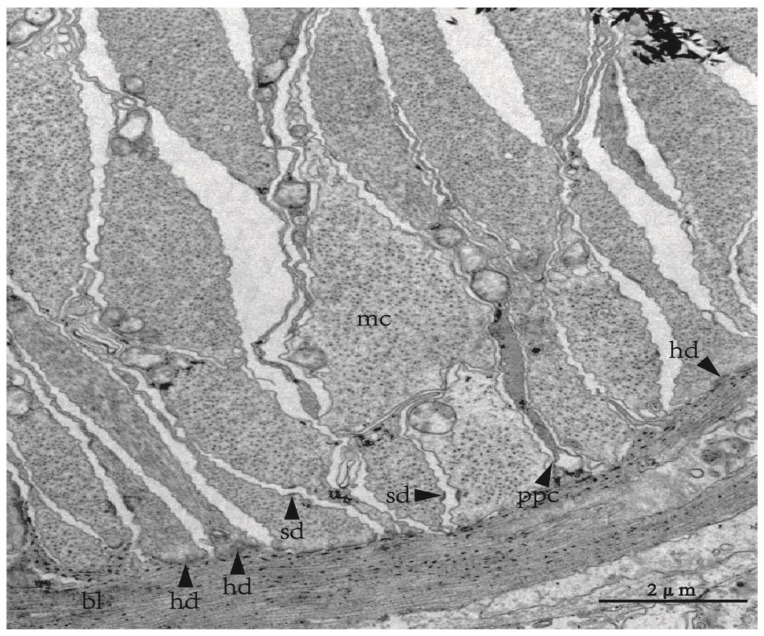
The myoepithelial organization in tube foot of *Apostichopus japonicus*. *Abbreviations*: bl, basal lamina; hd, hemidesmosome; mc, myoepithelial cell; ppc, process of peritoneal cell; sd, spot desmosome.

The tube feet and tentacles of echinoderms are body wall protuberances associated with the water vascular system, responsible for movement, adhesion, feeding, and sensation. Tube feet consist of a stem and terminal disk, with the former having a trilaminar organization consisting of an outer epidermis, a middle layer of connective tissue, and an inner celomic lining [32,33]. In all five classes of echinoderms, the celomic lining of the tube feet is a myoepithelium composed of two predominant cell types: peritoneal and myoepithelial cells. However, the morphology of the myoepithelium varies between different species from a simple myoepithelium in crinoids to a bipartite pseudostratified myoepithelium in echinoids and asteroids, with various intermediate organizations (simple or pseudostratified myoepithelium) in ophiuroids and holothurians [31,34,35,36,37]. Myoepithelial cells, consisting largely of thick and thin myofilaments, are orientated in parallel with the primary axis of tube feet and form a longitudinal muscular system, which is responsible for connecting the stem to the terminal disk [31]. The contraction of the longitudinal musculature can produce a backward pull, leading to a vacuum remaining between the basement of the tube feet and the surface of contact through the connective tissue, which facilitates adhesion [38]. The electron micrographs of myoepithelial organization in the tube feet can be seen in the figures of [31] and Figure 2.

The tentacles of holothurians can be divided into two major parts, the proximal stem and the distal branches. They are also composed of three major layers like tube feet: the epidermis, connective tissue, and celomic lining [33]. The water vascular canal of tentacles is formed by a myoepithelium where myoepithelial cells form a well-developed longitudinal muscle system within the tentacle that controls its movement. The myoepithelium of tentacles varies in different holothurians: there is a simple myoepithelium in *Holothuria forskali* [37] and *Leptosynapta tenuis* [31], and a pseudostratified myoepithelium in *Leptosynapta* spp. [39]. An electron micrograph of myoepithelial organization in the tentacles can be seen in the figures of [37].

#### 2.1.2. Musculature of the Internal Organs

The histological structure of the digestive tract in echinoderms consists of three layers: an inner digestive epithelium, a middle layer of connective tissue, and an outer celomic epithelium [28,40,41,42]. The celomic epithelium is usually a pseudostratified myoepithelium, where most myoepithelial cells are orientated perpendicular to the long axis of the gut, forming the outer circular musculature, and a small portion of myoepithelial cells parallel to the long axis constitutes the inner longitudinal musculature [28,41,43]. The myoepithelial cells in the longitudinal musculature are loosely arranged and partially embedded in the underlying connective tissue. Conversely, the myoepithelial cells in the circular musculature are clustered and continuously arranged, which can produce a more powerful contraction [41,44]. The distribution and development of these two muscle layers vary in different regions of the digestive tract. Typically, the circular layer occurs all along the digestive tract, while the longitudinal layer is scarcely visible in the anterior part and better developed in the posterior part [41,42,44]. The contraction of the circular and longitudinal musculature produces intestinal peristalsis and plays a basic role in the movement of ingested food [44]. Electron micrographs of the myoepithelial organization in the digestive tract can be seen in the figures of [28,41].

Besides the wall of the digestive tract, myoepithelial organization is also the common characteristic of the celomic epithelium in other internal organs, such as the ovaries [45], respiratory tree [42], and hemal system [31]. The ovaries of holothurians and crinoids have similar morphological detail and complexity, with both having a trilaminar structure: a germinal inner epithelium, connective tissue containing a genital hemal sinus, and an outer celomic epithelium. The outer celomic epithelium is a pseudostratified myoepithelium where myoepithelial cells form circular and longitudinal layers or only a circular layer [29,31,45,46,47]. However, in some holothurians, such as *Stichopus tremulus* and *Mesothuria intestinalis*, it is a simple myoepithelium that consists of only monociliated myoepithelial cells [48].

The ovaries of asteroids, ophiuroids, and echinoids have greater complexity, which includes an outer sac and an inner sac [47,49,50]. The organization of the inner sac is similar to and homologous to that of the ovaries of holothurians and crinoids, which consists of a germinal epithelium, connective tissue, and myoepithelium [45]. The outer sac, which is separated from the inner sac by a schizocoelic space, is also composed of three layers: an inner epithelium, a connective tissue layer, and an outer celomic epithelium. The inner epithelium is a myoepithelium in asteroids, a peritoneum in ophiuroids, and incomplete or virtually absent in echinoids [45,47,49,51]. And these muscles in ovaries mainly function to expel gametes by producing vigorous spawning contractions [46]. Electron micrographs of the myoepithelial organization in the ovaries can be seen in the figures of [29,46,49].

The respiratory trees of most holothurians are composed of an inner lining epithelium, a connective tissue layer, and an outer celomic epithelium. Myoepithelial cells in the celomic epithelium are orientated both along and perpendicular to the axis of the organ, forming a well-developed mesh of longitudinal and circular musculature. The contraction of these muscles contributes to respiratory movements together with other muscles of the body [30,42]. An electron micrograph of the myoepithelial organization in the respiratory tree can be seen in a figure in [42].

Holothuroids possess the most highly developed hemal system in echinoderms, where the wall of the hemal vessels typically has three layers: an outer epithelial layer, an intermediate circular muscle layer, and an inner connective tissue layer. The hemal vessels do not have obvious linings, and most of the lumen is occupied by connective tissue, forming a network. Epithelial and myoepithelial cells in the hemal vessel wall form a pseudostratified myoepithelium, which is anchored to a basal lamina, and most myoepithelial cells are arranged in a circular to oblique manner round the vessel. Nerve fibers are also present between the epithelial and myoepithelial cells [52,53]. An electron micrograph of a holothuroid hemal vessel can be seen in a figure in [52].

### 2.2. Somatic Musculature

**Figure 3:**
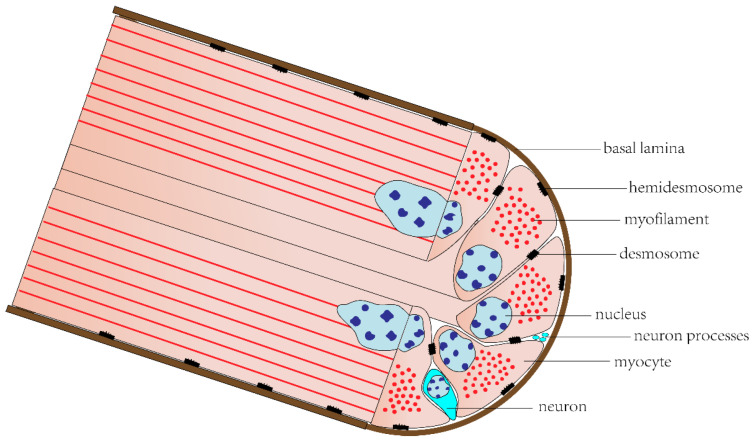
Muscle bundle of echinoderm somatic musculature.

Most echinoderms’ somatic musculature consists of large muscles with varying organization and function, which are composed of muscle bundles containing many myocytes [14]. These include longitudinal and circular muscles of the body wall in holothurians and asteroids [54,55], pharyngeal retractor muscles in holothurians [18], Aristotle’s lantern muscles in echinoids [56], arm muscles in ophiuroids and crinoids [57,58], and spine muscles in asteroids, echinoids, and ophiuroids [59,60,61]. Most of the cytoplasm of these myocytes is occupied by myofilaments, forming a powerful contractile apparatus. There are many myocyte processes extending into the center area of each muscle bundle, called “muscle tails”, which are responsible for receiving innervation. Each muscle bundle is surrounded by a basal lamina, to which myocytes are attached by hemidesmosomes, and adjacent myocytes are connected to each other by desmosomes. There are also neurons and their processes between myocytes (Figure 3) [14,25].

#### 2.2.1. Holothurian Somatic Musculature

- Longitudinal and circular muscles of the body wall

There are two muscle layers lining the body wall in holothurians: the inner is a circular muscle layer and the outer is a longitudinal muscle layer. Holothurians typically have five longitudinal muscles of the body wall (LMBW), which run along the longitudinal axis of the body and are connected to and innervated by the radial nerve via the mesentery. The circular muscle layer is located in the coelom lining of the body wall and interrupted by the LMBW and the radial nerve cords at each ambulacrum [21]. These two muscles work in coordination to regulate the animal’s movement. Myocytes of the LMBW have no well-developed sarcoplasmic reticulum (SR) but have many subsarcolemmal vesicles, which are considered potential storage sites for Ca^2+^ [25,54]. Furthermore, the LMBW has been regarded as the best model for studying excitation–contraction coupling in echinoderms due to its capacity for extreme stretch without physical damage [25]. An electron micrograph of the LMBW can be seen in a figure in [54].

- Pharyngeal retractor muscles

Holothurians of the order Dendrochirotida have five pharyngeal retractor muscles, which are connected to the calcareous ring at their anterior end and link with the LMBW at their posterior end. These are responsible for anchoring the oral complex (pharynx and buccal tentacles) to the body wall [18,62]. Myocytes of the pharyngeal retractor muscle are joined by desmosomes and arranged in muscle bundles surrounded by a basal lamina. The organization of these muscle bundles is uniform throughout the tissue. Functionally, the contraction of the pharyngeal retractor muscle brings about the retraction of the oral complex after it has been protracted for feeding [18,63]. An electron micrograph of the pharyngeal retractor muscle can be seen in a figure in [18].

#### 2.2.2. Echinoid Somatic Musculature

- Aristotle’s lantern muscles

Most echinoids possess a complex masticatory apparatus called Aristotle’s lantern, consisting of a muscular system and skeletal framework. The muscular system of Aristotle’s lantern is considered to be the most elaborate contractile system in echinoderms and includes seven major groups of muscles: pharyngeal levators and depressors, interpyramidal muscles, compass elevators and depressors, and lantern protractors and retractors [19,56]. Some of these muscles exhibit myoepithelial organization and others are muscle bundles [64].

Pharyngeal levators and depressors are an advanced form of bipartite pseudostratified myoepithelium, consisting of myoepithelial and peritoneal cells, where myoepithelial cells partly become immersed in the connective tissue and some peritoneal cells lose contact with the basal lamina. Interpyramidal muscles are a typical pseudostratified myoepithelium, and myoepithelial cells have well-developed myofilaments that make these muscles extremely powerful. Compass depressors and elevators function in respiratory movements and have different muscular structures. The myoepithelium on the adaxial surface of compass depressors is a bipartite pseudostratified myoepithelium. Myoepithelial cells are deeply immersed in the connective tissue and lie parallel to the longitudinal axis of the depressor. The myoepithelium on the oral surface of compass elevators is also a bipartite pseudostratified myoepithelium, but in the internal compartment, myocytes are arranged in typical muscle bundles. Protractors and retractors serve to push the lantern outwards and pull it back, respectively. The internal compartments of protractors and retractors are filled with muscle bundles surrounded by basal lamina. These muscle bundles are unevenly distributed in the connective tissue; most of them are abundant at the adaxial side of the muscle [56,64]. Electron micrographs of the lantern muscles can be seen in the figures of [56,64].

- Spine muscles

Three extant echinoderm classes (Asteroidea, Ophiuroidea, and Echinoidea) have calcified spines that are connected to the endoskeleton of the body wall through a mobile spine joint. The spine joint is the critical structure for spine movement, allowing the spine to incline and erect [59,60,61]. The spine joint of sea urchins is associated with a spine muscle and a ligament, or catch apparatus (CA), consisting of mutable collagenous tissue. The spine muscle runs longitudinally from the base of the spine to the test and surrounds the CA [60]. The CA consists mainly of collagen fibers but some smooth myocytes are also present, which run parallel to the collagen fibers, and both ends are attached to the collagen fibers [65]. The movement of the spine depends on the coordination of the spine muscle and CA, which bring about spine movement through the contraction of the spine muscle on one side, the relaxation of the spine muscle on the opposite side, and the destiffening of the whole CA [66].

#### 2.2.3. Asteroid Somatic Musculature

- Longitudinal and circular muscles of the body wall

Each arm of asteroids has two muscle layers at the celomic side of the dorsal body wall. The superficial layer is longitudinal muscle and the inner layer is circular muscle. The longitudinal muscle is thickened along the midline of the arm to form the apical muscle, which may contribute to upward arm flexion. The circular muscle layer is separated from the longitudinal muscle layer by connective tissue, which also divides the circular muscles into transverse muscle strands that insert into the body wall ossicles. The contraction of the circular muscles can produce a widening in the ambulacral groove and arm torsion [55,67]. A photomicrograph of the longitudinal and circular muscles of the body wall in asteroids can be seen in a figure in [67].

- Spine muscles

In asteroids, the morphology of the spine and the spine joint in the crown-of-thorns starfish is well studied. There are two groups of musculature in the joint; one is the musculature in the dermis surrounding the joint, and the other is the musculature that connects the spine and the pedicel. Both musculatures are smooth and oriented along the long axis of the spine. The distribution of these muscles suggests that they permit a passive inclination of the spine and bring about its active erection [59].

#### 2.2.4. Ophiuroid Somatic Musculature

- Arm muscles

The arms of ophiuroids consist of a series of homonomous segments. Each segment contains (1) an internal vertebral ossicle, the vertebral ossicles of adjacent segments connected by an intervertebral ligament and paired oral and aboral intervertebral muscles, and (2) four external arm plates: oral, aboral, and paired lateral plates. These intervertebral muscles are attached to special areas of the vertebral ossicle. The contraction of these intervertebral muscles causes adjacent ossicles to rotate around the intervertebral joint, which controls arm movement [57,68].

- Spine muscles

The spines of ophiuroids are connected to the lateral arm plate by the spine muscle and ligament. The spine muscle consists of two muscle bundles of myocytes, which originate in different areas of the lateral arm plate and converge to form a single bundle at the base of the spine. The morphology of the two muscle bundles varies widely across the class, this being smooth muscle in *Ophiothrix fragilis* and *Amphipholis squamata* and obliquely striated muscle in *Ophiocomina nigra*, where adjacent myofilaments are progressively staggered. The contraction of the muscle is mainly responsible for erecting the spine [61,69,70]. An electron micrograph of the spine muscle in ophiuroids can be seen in a figure in [61].

#### 2.2.5. Crinoid Somatic Musculature

- Arm muscles

Crinoid arms consist mainly of a series of brachial ossicles that are linked at mobile joints. Adjacent brachial ossicles are connected by a pair of flexor muscles [58]. The flexor muscle bundles consist of different obliquely striated fibers (A-type and B-type fibers) and some rare smooth fibers (C-type fibers). A- and B-type fibers exhibit different myofilament arrangements and distribution. A-type fibers are present along the whole arm and form the central mass of the muscle bundle. B-type fibers only develop in the middle and proximal parts of the arm, spread more peripherally, and partly or completely surround the central mass. In addition, there are C-type fibers randomly distributed along the edge of the flexor muscle bundle or in the midzone between A- and B-type fibers. These three different muscle fibers are responsible for performing different types of arm movements, with A-type fibers performing fast movements, B-type fibers performing powerful actions, and C-type fibers related to slow movements [71,72,73]. Electron micrographs of the arm muscle in crinoids can be seen in the figures of [72].

## 3. Neurotransmitters That Affect Echinoderm Muscles

### 3.1. Cholinergic Neurotransmitters

We summarize the effects of various neurotransmitters on different muscles of echinoderms (Table 1). In the neuromuscular systems of echinoderms, the typical excitatory cholinergic neurotransmitter is acetylcholine (ACh), which induces contractile responses in various muscles in echinoderms [74]. In holothurians, Ach induces the contraction of the LMBW, intestinal muscle, cloaca, tube feet, and tentacles in different concentration ranges [75,76]. In *A. japonicus*, ACh leads to an initial sharp and tonic contraction of the LMBW, followed by very gradual relaxation [75]. ACh-induced intestinal contraction is observed in both longitudinal intestine strips and intestine rings, suggesting that both the longitudinal and circular muscles within the intestinal muscle layer can be activated by ACh [76]. In asteroids, ACh induces the contraction of the apical muscle, tube feet, and cardiac stomach. But the contraction of the cardiac stomach by ACh is too brief to measure the effect of relaxing agents, so in many pharmacological experiments, a high-K^+^ solution was used to induce a sustained contraction of the cardiac stomach [15,77,78,79]. In addition, ACh was detected in the ovaries and testes of the starfish *Patiria pectinifera* and induced the contraction of the gonadal wall to promote ovulation [20]. In echinoids, ACh can induce the contraction of the spine muscle, lantern retractor and protractor muscle, compass elevator muscle, tube feet, and radial muscle (echinothuriid) [80,81,82,83,84]. The subepithelial nerve plexus of the tube feet contains conspicuous amounts of ACh, which is released from nerve terminals of the subepithelial nerve plexus and diffuses through the connective tissue layer to reach the muscle layer [83]. However, ACh does not have an excitatory effect in all echinoderm muscles; for example, it cannot stimulate contraction and causes a decrease in tone of the brachial muscles in the arms of the feather star *Antedon mediterranea* [58]. 

There are many factors that influence ACh-induced contraction, such as the external Ca^2+^ concentration; Mn^2+^, which blocks Ca^2+^ influx in many excitable membranes; and procaine, which inhibits Ca^2+^ release from the sarcoplasmic reticulum (SR) [75,95,96]. The LMBW contraction induced by ACh completely disappears in a Ca^2+^-free solution and the degree of contraction is significantly reduced by procaine. It can also be markedly enhanced after the mechanical response to Na^+^ removal, which may be due to the Na^+^-Ca^2+^ exchange mechanism that increases intracellularly stored Ca^2+^ by allowing Ca^2+^ influx and inhibiting its efflux [75]. These results suggest that the degree of ACh-induced contraction depends not only on the gradient of external Ca^2+^ across the plasma membrane but also the release of intracellularly stored Ca^2+^. 

ACh acts through acetylcholine receptors (AChRs), which can be divided into nicotinic receptors (nAChRs) and muscarinic receptors (mAChRs) in deuterostomes and some protostome groups based on different pharmacological properties [91,97,98]. Pharmacological evidence suggests that nAChRs and mAChRs are co-localized in numerous echinoderm muscles and mediate different responses, respectively. When the LMBW is stimulated to contract by ACh, nAChRs are responsible for modulating muscle tone and mAChRs are responsible for initiating and enhancing rhythmicity [74,97]. Furthermore, ACh was shown to stimulate ovulation in *P. pectinifera* through activating mAChRs because it was inhibited by a mAChR antagonist, and a nAChR antagonist had no effect on it [20]. These results indicate that the two receptors have different signaling pathways. In fact, the nAChR is a pentameric subunit-assembled ion channel. In vertebrates, 17 nAChR subunits have been identified, and different subunit combinations show different pharmacological properties [98,99,100]. At the echinoderm neuromuscular junction, distinct subpopulations of nAChR have been identified in pre- and postsynaptic positions by using specific neuronal nAChR antagonists and muscle nAChR antagonists [74,97]. In the *Strongylocentrotus purpuratus* genome, at least 12 nAChR subunit-encoding genes have been identified [13]. As an ionotropic receptor, nAChR can directly gate ion channels without second messengers [101]. During the fertilization of sea urchin eggs, the activation of nAChR gated inward Na^+^ channels and caused depolarization by Na^+^ influx [100]. The mAChR is a member of the rhodophosin-like G-protein-coupled receptor (GPCR) family and acts through coupling G-proteins rather than gating ion channels directly. Therefore, the responses of mAChRs are very delayed compared with those of nAChRs [98]. Five mAChR subtypes (M1-M5) have been identified in vertebrates, with coupling to different G-proteins [98,102]. M1, M3, and M5 receptors are associated with G_q_/G_11_ and activate intracellular Ca^2+^ mobilization through the phosphoinositol signaling pathway. M2 and M4 receptors are associated with G_i_/G_o_ and maintain stable intracellular Ca^2+^ levels by reducing protein kinase A activity [98,103,104,105,106]. Distinct mAChR subtypes are also present in echinoderm muscles, which share some pharmacological characteristics with M1-M5 receptors and may be their precursors [74,97]. In the LMBW of *Sclerodactyla briareus*, by using a series of M1-M5 agonists and antagonists, the presence of M1-, M3-, M5- and M2-, M4-like receptors has been identified [106]. Furthermore, pharmacological evidence suggests that the signaling pathway of mAChR activation in echinoderms is similar to that in vertebrates. For example, the LMBW contraction induced by M1, 3, and 5 receptor agonist (oxotremorine M) was blocked by LiCl treatment, an inhibitor of the phosphoinositol signaling pathway [106]. The M2/M4 antagonist methoctramine accelerated the relaxation after ACh-induced contraction, which implied a rapid decline in intracellular Ca^2+^ levels [97].

Although pharmacological experiments have indicated that these echinoderm AChR subtypes show similar pharmacological properties to vertebrate counterparts to some extent, due to the poor selectivity of some antagonists and agonists to echinoderm muscles and the co-expression of distinct receptor subtypes, it is difficult to accurately identify the distinct receptor subtypes and distinguish their respective functions in echinoderms [97,98]. Another approach to identify the distinct functions of AChRs is gene knockout, which has been used in mammals to describe the functional characteristics of some AChR subtypes. It would be interesting to use gene knockout or knockdown to identify the function of AChRs in echinoderms as well [107,108].

### 3.2. Bioamine Neurotransmitters

Many bioamine neurotransmitters, such as 5-HT, dopamine, adrenaline, and noradrenaline, have been identified in different tissues of echinoderms using biochemical and histochemical methods [21,44,109,110]. These neurotransmitters are involved in regulating a variety of physiological behaviors in echinoderms, including reproduction, ovarian maturation, embryonic development, morphogenesis, settlement, and swimming behavior [21,93,110,111,112,113]. At the same time, the effects of numerous bioamines on various echinoderm muscles have been examined, and their functional characteristics are highly variable [21,86]. 

In holothurians, 5-HT, dopamine, adrenaline, noradrenaline, and DOPA had no effects on the LMBW, but 5-HT had an inhibitory effect on ACh-induced contraction [21]. On the isolated cloaca preparation, 5-HT caused a contraction similar to that induced by ACh, but dopamine, adrenaline, and noradrenaline produced variable results or no effects that could be distinguished from spontaneous activities of the cloaca [85]. In asteroids, adrenaline, noradrenaline, dopamine, and histamine all had effects on tube feet. Adrenaline was five times more effective at inducing tube foot contraction than noradrenaline and ten times more effective than dopamine. However, at concentrations higher than 10^−5^ M, adrenaline and noradrenaline induced slow relaxation instead of contraction. This biphasic effect also occurred in skinned tube feet, suggesting that it influenced the muscle layer rather than the mechanical properties of the connective tissue. Histamine induced the relaxation of both intact and skinned tube feet. 5-HT had no effect on tube feet, even at 10^−4^ M [86]. In echinoids, the effects of bioamines on the lantern retractor muscle, spine muscle, tube feet, and esophageal muscle have been examined. Noradrenaline and dopamine caused a rapid relaxation of the lantern retractor muscle, and sometimes initiated long-lasting rhythmic activities [89]. Adrenaline caused relaxation and inhibited spontaneous activities and ACh-induced contraction [80]. Octopamine caused spine muscle contraction, while dopamine and noradrenaline relaxed the contraction elicited by octopamine and ACh [66]. In the larva of echinoids, 5-HT and dopamine had a strong stimulatory effect on esophageal muscle activity [109, 110]. However, no bioamines have been found to affect the tube foot muscle [88]. In crinoids, bioamines either failed to activate any responses in muscles or produced inconsistent effects [16,58].

Bioamine neurotransmitters function by activating their receptors, such as 5-HT receptors (5-HTRs), dopamine receptors, and adrenergic receptors [111,114,115]. In echinoderms, the genes encoding these receptors and the localization of some receptors have been discovered by means of genomic and immunochemical methods [13,111,114,116]. These receptors play an important role in regulating various behaviors in echinoderms. For example, dopamine receptors regulate the growth rate of the larval post-oral arms and righting behavior in *S. purpuratus* [115,117], and 5-HTRs regulate movement and respiratory metabolism in *A. japonicus* [111,114]. In mammals, the signaling pathway of these receptors has been reported in detail, including the activation of typical intracellular signaling cascades, such as G-proteins, adenylate cyclase, and phospholipase C, and altering the permeability of ion channels [118,119]. However, few studies on the signaling pathway mediated by bioamine receptors have been reported in echinoderms, especially regarding muscle regulation. Limited research indicated that 5-HT inhibited ACh-induced LMBW contraction in *A. japonicus* [21], and a further study found that 5-HT_4/6_ is highly expressed in the LMBW, which is a rhodopsin-type GPCR and mediates intracellular cAMP accumulation by coupling G_s_ protein [111]. However, the relationship between these receptor subtypes and the LMBW contraction process was not clear and further experiments are needed to identify the specific signaling pathway.

### 3.3. Amino Acid Neurotransmitters

The investigation of echinoderm muscle pharmacology has included many amino acid neurotransmitters, such as gamma-aminobutyric acid (GABA), glutamate, and glycine. However, only GABA has a relatively obvious role and has been extensively studied [86,88,91]. Therefore, the effects of GABA on the muscles of echinoderms are mainly discussed here. Immunocytochemical studies have indicated that GABA is mainly localized in nervous and muscular systems in echinoderms and plays an important role in the settlement of echinoderm larva [74,93,120,121,122]. Pharmacological evidence suggests that GABA has different effects on echinoderm muscles: an excitatory effect in asteroids and echinoids and an inhibitory effect in holothurians [74,86,88,120].

In asteroids and echinoids, GABA induced tube feet contraction, reaching 70-80% of that produced by ACh [86,88,92]. In holothurians, GABA was reported to cause LMBW relaxation, inhibit the rhythmicity of spontaneous contraction, reduce ACh-induced contraction, and decrease the amplitude of spontaneous cloaca contraction [85,91]. In crinoids, L-glutamate could induce rhythmic muscle contraction on the arm muscles, whereas no effect was detected for GABA and ACh, suggesting that L-glutamate may be the main excitatory neurotransmitter in crinoids [58]. 

GABA receptors (GABARs) can be divided into two types: GABA_A_Rs and GABA_B_Rs [74,123]. GAB_A_ARs are pentameric ligand-gated anion-selective channels and GABA_B_Rs are heterodimeric G-protein-coupled receptors [123]. In vertebrates, GABA_A_Rs mediate hyperpolarization by inducing chloride ion inflow, thereby reducing cell excitability [124], whereas in echinoderms, GABA_A_Rs mediate the excitatory responses and induced depolarization of tube feet muscle preparations by enhancing membrane permeability to Na^+^ [74,88,120]. Furthermore, cholinergic receptor blockers and cholinesterase inhibitors can inhibit and potentiate GABA excitatory responses in sea urchin tube feet. Therefore, Florey et al. (1975) speculated that GABA does not act directly on tube feet muscle but through the cholinergic system [88]. In vertebrates, GABA_B_Rs induce hyperpolarization by activating the G protein G_i/o_, inhibit voltage-gated Ca^2+^ channels, or activate K^+^ channels [125]. As in vertebrates, GABA_B_Rs in echinoderms also mediate inhibitory responses [74,91]. However, the signaling pathway mediated by GABA_B_Rs in echinoderms remains to be studied.

### 3.4. Gaseous Neurotransmitters

It has been demonstrated that ACh is the major excitatory transmitter in the echinoderm neuromuscular system. However, a universal inhibitory neurotransmitter has not been identified. Neurotransmitters such as 5-HT, GABA, and dopamine all have diverse effects on echinoderm muscles. Nitric oxide (NO) is a class of gaseous neurotransmitters, which is increasingly found to play important signaling functions in many organisms [126]. In echinoderms, NO is involved in regulating reproduction, the stress response, muscle activity, and larval settlement and metamorphosis [127,128,129,130]. It has a consistent relaxation effect in many muscle preparations, including the cardiac stomach, tube feet, and apical muscle in asteroids and the pyloric sphincter in echinoids [94,127,131]. Therefore, NO may be a universal muscle relaxant in echinoderms [15]. 

Unlike conventional neurotransmitters that are released from vesicles and function by binding to membrane receptors, NO, as a membrane-permeant molecule, enters the target cell by free diffusion [126]. It is formed by nitric oxide synthases (NOSs) using L-arginine as a substrate [132]. In mammals, based on the different cell types, NOSs can be divided into three types: endothelial NOS (eNOS), neuronal NOS (nNOS), and inducible NOS (iNOS) [133]. In most invertebrates, however, only one *NOS* gene has been identified [134,135]. In asteroids, pharmacological experiments showed that L-arginine could induce cardiac stomach relaxation, suggesting the presence of NOS in this tissue [15]. In echinoids, nNOS-positive neuron-like cells were detected at the pylorus, which could produce NO to regulate the pyloric sphincter [94]. In target cells, NO activates soluble guanylyl cyclase (SGC) and causes guanosine 3′,5′-cyclic monophosphate (cGMP) accumulation, and this NO-SGC-cGMP signaling pathway is highly conserved in diverse organisms [136]. Applying the starfish cardiac stomach as a model, combined with some pharmacological inhibitors, Elphick and Melarange (2001) demonstrated the existence of the NO-SGC-cGMP pathway in an echinoderm. In this model, NO derived from the basiepithelial plexus of the starfish cardiac stomach diffuses across the connective tissue layer into the muscle cell, leading to cGMP accumulation by activating SGC [15]. In mammals, NO-induced cGMP accumulation mediates the activation of cGMP-dependent protein kinase (PKG), which then decreases IP_3_ production, activates sarcoplasmic/endoplasmic reticulum Ca^2+^ ATPase, and regulates myosin light-chain phosphatase. This pathway induces muscle relaxation by reducing the intracellular Ca^2+^ level and Ca^2+^ sensitivity [137,138]. However, the mechanism of cGMP-mediated muscle relaxation in echinoderms remains unclear and more experiments are needed to determine the universality of this pathway in echinoderm muscles.

## 4. Neuropeptides

Neuropeptides are an important class of signaling molecules in organisms that are widely involved in and regulate various physiological behaviors. They are derived from larger precursor proteins that are synthesized and secreted by neurons [139]. In echinoderms, a variety of neuropeptides have been predicted through genomic, transcriptomic, and proteomic analysis that have been suggested to be involved in regulating important physiological processes such as feeding, reproduction, growth, and muscle activity [22,140,141,142,143]. The first neuropeptide identified in echinoderms was a SALMFamide, which caused the relaxation of various muscles, such as the cardiac stomach, apical muscle, and tube feet in asteroids and the intestine and LMBW in holothurians [27,144,145,146]. Subsequently, an increasing number of neuropeptides in echinoderms have been investigated using pharmacological methods for their regulatory effects on muscles (Table 2). Some neuropeptides exhibit conserved muscle-regulatory function in different echinoderms, such as Pedal peptide/orcokinin-type neuropeptides, Luqin-type neuropeptides, and Calcitonin-type neuropeptides [78,79,147,148]. However, some neuropeptides, such as Vasopressin/oxytocin-type neuropeptides, exhibit the opposite effect in different echinoderms, inducing relaxation in asteroids and contraction in holothurians and echinoids [26,149]. Studies of the phylogenetic distribution of neuropeptides and their homologous receptors in different species contribute to understanding the evolutionary history of related neuropeptide families. Echinoderms, occupying an “intermediate” phylogenetic position between chordates and protostomes, are “important links” for reconstructing these evolutionary histories. For example, Somatostatin-type (SS) and allatostatin-C-type (ASTC) neuropeptides, which were identified in chordates and protostomes, respectively, both exert inhibitory effects on muscles. However, echinoderms have two SS/ASTC-type neuropeptides, SS1 and SS2, that are the orthologs of ASTC and SS, respectively, and exert completely opposite muscle regulation effects: SS-type neuropeptides cause relaxation and ASTC-type neuropeptides cause contraction [23]. This functional difference between SS/ASTC neuropeptides in echinoderms provides new insights into reconstructing the evolutionary history of this neuropeptide family.

There are two noticeable problems in the muscle regulation of echinoderm neuropeptides. The first is the structure–activity relationship of neuropeptides. Some neuropeptides from the same neuropeptide family or the same precursor exhibit different potency, such as SALMFamide-2 (S2, SGPYSFNSGLTFamide) and SALMFamide-1 (S1, GFNSALMFamide) in *A. rubens*. Pharmacological experiments have shown that S2 has greater relaxation potency than S1, and structure–activity relationship studies of SALMFamides have suggested that the four additional N-terminal residues of S2 may be associated with neuropeptide activity because it can promote the self-association of S2 and allow S2 to form a structured conformation in aqueous solution, but S1 cannot [160]. The second is the tissue-specific effects of neuropeptides on different muscles from the same organism. In *A. rubens*, NGFFYamide has an inhibitory effect on apical muscle but an excitatory effect on tube feet [153]. Our research also suggested that Pedal peptide/orcokinin-type neuropeptides had a significant relaxation effect on the intestine of *A. japonicus* but almost no effect on LMBW [158]. So, further identification of neuropeptide receptors and clarification of the signaling pathway will help us to figure out the regulation mechanism of neuropeptides on muscle contraction/relaxation.

Typically, neuropeptides function by binding to G-protein-coupled receptors (GPCRs), causing signal transmission at the cellular level through second messengers, which later leads to physiological changes at the tissue/organism level [161]. So far, many GPCRs have been identified and cloned in echinoderms, and signaling pathways downstream of some receptors have been preliminarily explored, including Ca^2+^ mobilization, cAMP accumulation, and ERK1/2 phosphorylation [26,78,148]. However, the complete molecular and cellular mechanisms by which neuropeptides control muscle contraction/relaxation and the differences in regulatory mechanisms between different neuropeptides are still unknown. In schistosomes and mammals, detailed intracellular signaling cascades and membrane channel activation of neuropeptide-induced muscle contraction have been reported, which can provide guidance for related research on echinoderms [162,163].

Based on current studies of myoactive neuropeptides in echinoderms and other species, we speculated that there are three potential mechanisms mediating muscle regulation by neuropeptides in echinoderms: (1) Neuropeptides act directly on specific GPCRs located on muscle cells to control membrane channel activity through signaling cascades mediated by intracellular second messengers, thereby causing muscle contraction/relaxation. For example, Alzugaray et al. (2021) found that the allatotropin-type neuropeptide (AT) regulated muscle contraction through the AT receptor/G_q_/PLC/IP_3_/intracellular Ca^2+^ signaling pathway in *Hydra* [164]. In addition, by using blockers of voltage-gated calcium channels (VGCCs), they found that extracellular Ca^2+^ influx through VGCCs also participates in muscle contraction [165]. In *A. japonicus*, we found that the vasopressin/oxytocin-type neuropeptide (VP/OT) has a strong contractile effect on a variety of muscle tissues, including LMBW, intestinal muscle, and tentacles. Ca^2+^ deprivation experiments have shown that VP/OT-induced contraction is dependent on extracellular Ca^2+^ influx [150]. However, the channel that mediates Ca^2+^ influx and the intracellular signaling pathway that activates it are still unknown. (2) Neuropeptides exert a myoexcitatory effect by directly activating peptide-gated cation channels on muscle cells. For example, Schmidt et al. (2018) found a peptide-gated ion channel in the marine annelid *Platynereis dumerilii* that is activated by myoinhibitory peptides (MIPs) and belongs to the degenerin/epithelial Na^+^ channel (DEG/ENaC) family [166]. A previous study showed that MIPs can function by activating a specific GPCR [166,167]. These results suggest that neuropeptides may have a dual signaling system in some organisms, transmitting distinct signaling through both ionotropic and metabotropic receptors [166]. However, no peptide-gated ion channels have been identified in echinoderms; the neuropeptides reported so far all function by activating metabotropic GPCRs. (3) Neuropeptides control muscle contraction/relaxation by activating other neurons that secrete other neurochemicals. In *Caenorhabditis elegans*, neuropeptide-like protein 40 induced GABAergic neurons to release γ-GABA by binding with a specific receptor located on GABAergic neurons to control the rhythmic contraction of intestinal muscle [168]. In echinoderms, Odekunle et al. (2019) were the first to report the anatomical expression patterns of the VP/OT-type neuropeptide and its receptor in the starfish *A. rubens* using double-labelling fluorescence immunohistochemistry. Based on the expression of the receptor in the basiepithelial neural plexus, they speculated that VP/OT-type neuropeptides may indirectly regulate muscle relaxation by participating in other neural processes [26]. Elphick and Melarange (2001) also reported the relationship between SALMFamides and NO, which both induced cardiac stomach relaxation in starfish. However, SALMFamides do not function through NO, and they belong to different neural signaling systems [15]. So far, in echinoderms, no conclusive evidence has been provided that shows neuropeptides can mediate muscle contraction/relaxation through other neurons.

## 5. Conclusions

The musculature of echinoderms includes the myoepithelium and muscle bundles. The myoepithelium is composed mainly of myoepithelial and peritoneal cells and can be divided into different types according to the relative position of these cells, including the simple myoepithelium, pseudostratified myoepithelium, bipartite pseudostratified myoepithelium, and stratified myoepithelium. Muscle bundles are formed by the aggregation of several myocytes surrounded by basal lamina, most of which consist of smooth muscle fibers, but obliquely striated muscle fibers are also present in ophiuroids and crinoids. Pharmacological experiments have demonstrated the effects of many classic neurotransmitters on echinoderm muscles, of which ACh and NO are considered to be the main excitatory and inhibitory neurotransmitters in echinoderms, respectively. However, other classic neurotransmitters, such as 5-HT, dopamine, adrenaline, noradrenaline, GABA, and L-glutamate, have variable effects on different muscles. In recent years, an increasing number of neuropeptides have also been identified to have muscle-regulating effects in different echinoderm species. Neurotransmitters and neuropeptides usually function through specific receptors, and although many receptors have been identified in echinoderms, the complete downstream signaling pathways of these receptors are unknown. Studies on how these neurotransmitters induce muscle contraction/relaxation will help to understand the potential mechanisms of muscle regulation. Echinoderms have a deuterostome mode of development, which results in their muscle physiology having more in common with that of vertebrates than that of other invertebrates. Therefore, studies on the regulatory mechanisms of echinoderm muscle systems will provide new insights into the evolution of regulatory systems in deuterostome muscle.

## Figures and Tables

**Table 1:** The effect of classic neurotransmitters on various echinoderm muscles. LMBW, longitudinal muscle of body wall; IM, intestinal muscle; TF, tube foot; TM, tentacle muscle; CA, cloaca; AM, apical muscle; CS, cardiac stomach; EM, esophageal muscle; LRM, lantern retractor muscle; ME, myoexcitatory effect; MI, myoinhibitory effect; NE, no effect.

Neurotransmitters	Holothuroidea					Asteroidea			Echinoidea		
	LMBW	IM	TF	TM	CA	AM	CS	TF	EM	TF	LRM
ACh	ME [54]	ME [76]	-	-	ME [85]	ME [79]	ME [15]	ME [86]	ME [87]	ME [88]	ME [89]
5-HT	MI [21]	-	-	-	ME [85]	-	-	NE [86]	ME [87]	NE [88]	NE [81]
Dopamine	NE [21]	-	-	-	MI [85]	-	-	ME [86]	ME [87]	NE [88]	MI [89]
Adrenaline	NE [21]	-	-	-	NE [85]	-	-	ME/MI [86]	-	-	MI [80]
Noradrenaline	NE [21]	-	-	-	NE [85]	-	-	ME/MI [86]	-	NE [83]	MI [89]
Tryptamine	-	-	-	-	MI [85]	-	-	-	ME [90]	-	NE [81]
Histamine	-	-	-	-	-	-	-	MI [86]	-	NE [83]	NE [81]
GABA	MI [91]	-	-	-	MI [85]	-	-	ME [86]	NE [92]	ME [88]	MI [80]
L-glutamate	-	-	-		ME/MI [85]	-	-	ME [86]	-	NE [83]	NE [81]
Glycine	-	-	-	-	NE [85]	-	-	NE [93]	-	NE [87]	-
Nitric oxide	-	-	-	-	-	MI [27]	MI [27]	MI [27]	MI [94]	-	-

**Table 2:** The effect of neuropeptides on various echinoderm muscles. LMBW, longitudinal muscle of body wall; IM, intestine muscle; TM, tentacle muscle; AM, apical muscle; CS, cardiac stomach; EM, esophageal muscle; ME, myoexcitatory effect; MI, myoinhibitory effect; NE, no effect.

Neuropeptide	Holothuroidea			Asteroidea			Echinoidea	
	LMBW	IM	TM	AM	CS	TF	EM	TF
Vasopressin/oxytocin-type	ME [150]	ME [150]	ME [150]	MI [26]	MI [26]	NE [26]	ME [149]	ME [149]
Calcitonin-type	-	-	-	MI [79]	NE [79]	MI [79]	-	-
Luqin-type	MI [148]	NE [148]	-	-	NE [147]	MI [147]	-	-
L-type SALMFamides	MI [145]	MI [145]	-	MI [27]	MI [146]	MI [27]	-	-
F-type SALMFamides	NE [151]	MI [151]	-	MI [27]	MI [146]	MI [27]	-	-
NG-type	ME [152]	MI [152]	ME [152]	MI [153]	ME [154]	ME [153]	ME [149]	ME [149]
Somatostatin/allatostatinC-type1	-	-	-	ME [23]	ME [23]	ME [23]	-	-
Somatostatin/allatostatinC-type2	-	-	-	NE [155]	MI [155]	MI [155]	-	-
Sulfakinin/cholecystokinin-type	-	MI [76]	-	ME [156]	ME [156]	ME [156]	-	-
Gonadotropin-releasing hormone type	-	-	-	ME [157]	ME [157]	ME [157]	-	-
Corazonin	-	-	-	ME [157]	ME [157]	ME [157]	-	-
Pedal peptide/orcokinin-type1	-	-	-	MI [78]	MI [78]	MI [78]	-	-
Pedal peptide/orcokinin-type2	NE [158]	MI [158]	-	NE [159]	MI [159]	NE [159]	-	-

## Data Availability

Not applicable.
